# Dysregulated Prefrontal Cortex Inhibition in Prepubescent and Adolescent Fragile X Mouse Model

**DOI:** 10.3389/fnmol.2020.00088

**Published:** 2020-05-26

**Authors:** Ioannis Kramvis, Rhodé van Westen, Hanna C. A. Lammertse, Danai Riga, Tim S. Heistek, Alex Loebel, Sabine Spijker, Huibert D. Mansvelder, Rhiannon M. Meredith

**Affiliations:** ^1^Department of Integrative Neurophysiology, Center for Neurogenomics and Cognitive Research, Vrije Universiteit, Amsterdam, Netherlands; ^2^Department of Functional Genomics, Center for Neurogenomics and Cognitive Research, Vrije Universiteit, Amsterdam, Netherlands; ^3^Department of Molecular and Cellular Neurobiology, Center for Neurogenomics and Cognitive Research, Vrije Universiteit, Amsterdam, Netherlands; ^4^Department of Neurobiology, Ludwig-Maximilians Universitat, Munich, Germany

**Keywords:** Fragile X, prefrontal cortex, GABA, electrophysiology, plasticity

## Abstract

Changes in excitation and inhibition are associated with the pathobiology of neurodevelopmental disorders of intellectual disability and autism and are widely described in Fragile X syndrome (FXS). In the prefrontal cortex (PFC), essential for cognitive processing, excitatory connectivity and plasticity are found altered in the FXS mouse model, however, little is known about the state of inhibition. To that end, we investigated GABAergic signaling in the Fragile X Mental Retardation 1 (FMR1) knock out (Fmr1-KO) mouse medial PFC (mPFC). We report changes at the molecular, and functional levels of inhibition at three (prepubescence) and six (adolescence) postnatal weeks. Functional changes were most prominent during early postnatal development, resulting in stronger inhibition, through increased synaptic inhibitory drive and amplitude, and reduction of inhibitory short-term synaptic depression. Noise analysis of prepubescent post-synaptic currents demonstrated an increased number of receptors opening during peak current in Fmr1-KO inhibitory synapses. During adolescence amplitudes and plasticity changes normalized, however, the inhibitory drive was now reduced in Fmr1-KO, while synaptic kinetics were prolonged. Finally, adolescent GABA_A_ receptor subunit α2 and GABA_B_ receptor subtype B1 expression levels were different in Fmr1-KOs than WT littermate controls. Together these results extend the degree of synaptic GABAergic alterations in FXS, now to the mPFC of Fmr1-KO mice, a behaviourally relevant brain region in neurodevelopmental disorder pathology.

## Introduction

Deregulated excitatory/inhibitory balance is proposed to underlie neuronal dysfunction and cognitive impairments in neurodevelopmental disorders of autism and intellectual disability, including Fragile X syndrome (FXS; Rubenstein and Merzenich, 2003; Gibson et al., 2008; Lozano et al., 2014; Contractor et al., 2015). Caused by silencing of the Fragile X Mental Retardation 1 (FMR1) gene, FXS is characterized by intellectual disability with high concomitance for autism spectrum disorders, epilepsy, attentional, executive control and behavioral deficits (Munir et al., 2000a,b; Wilding et al., 2002; Sullivan et al., 2006; Lozano et al., 2014). Central to many deficits presented in FXS is the prefrontal cortex (PFC), known to coordinate high order cognitive processes (Miller and Cohen, 2001). PFC dysfunction is associated with several neurodevelopmental and neuropsychiatric disorders (Arnsten and Rubia, 2012). In medial PFC (mPFC) of FXS mice, excitatory synaptic function, connectivity, and plasticity are altered (Meredith et al., 2007, 2012; Krueger et al., 2011; Testa-Silva et al., 2012). However, little is currently known about prefrontal inhibitory synaptic function and plasticity in FXS, particularly during early postnatal and pre-adulthood periods.

Fragile X Mental Retardation Protein (FMRP), binds, traffics, and translationally regulates mRNAs, at pre- and post- synaptic sites (Bramham and Wells, 2007; Christie et al., 2009; Darnell et al., 2011; Akins et al., 2012). Importantly, FMRP has been shown to bind both GABA_B_ receptor subunit mRNAs (Wolfe et al., 2016), and to at least GABA_A_ receptor subunit δ and α1 mRNA (Braat et al., 2015). Additionally, positron emission tomography in FXS patients demonstrated a significant reduction of GABA_A_ receptor ligand-binding potential throughout the brain (D’Hulst et al., 2015). Furthermore, in autistic patients (Brondino et al., 2016), and FXS mouse model GABAergic changes occur across multiple brain regions, including cortex, amygdala, striatum, and hippocampus (El Idrissi et al., 2005; D’Hulst et al., 2006; Selby et al., 2007; Centonze et al., 2008; Paluszkiewicz et al., 2011; Vislay et al., 2013; Modgil et al., 2019). Finally, the post-synaptic deletion of FMRP is sufficient to prolong inhibitory decay kinetics, while global FMRP deletion also impacts synaptic GABA levels and neurotransmitter clearance (Vislay et al., 2013). Thus, FMRP is critical for proper inhibitory synaptic communication, both at pre- and postsynaptic inhibitory domains.

GABAergic inhibition undergoes significant maturation during at least the first month of postnatal development (Deidda et al., 2014). During then, rodent hippocampal and cortical inhibitory kinetics quicken accompanied by GABA subunit expression changes, frequency of inhibition gradually increases to adult levels, while amplitudes remain fairly stable (Dunning et al., 1999; Banks et al., 2003). Changes caused by FMRP deletion are not always persistent, and in many cases are found to be transient or shifted in developmental time (Meredith et al., 2012; Contractor et al., 2015). For example, In Fmr1-KO amygdala, spontaneous inhibitory frequency persistently deviates from control values during the first month of postnatal development, while spontaneous inhibitory decay kinetics transiently normalize during the 2nd and 3rd postnatal weeks (Vislay et al., 2013). Consequently, the deletion of FMRP appears to impact inhibitory signaling in a dynamic and complex manner.

While inhibition mostly matures by adolescence (Le Magueresse and Monyer, 2013; Gonzalez-Burgos et al., 2015), prefrontal rodent and human development carries through (summarized in de Almeida et al., 2013), and is shown altered in adolescent FXS patients (Menon et al., 2004; Hoeft et al., 2007; Bray et al., 2011). In this study, we provide the first evidence for changes in GABAergic inhibition during the 3rd (prepubescence) and 6th (adolescence) postnatal weeks in Fmr1-KO mPFC. Prepubescent synaptic inhibitory drive and amplitudes were increased. In adolescence, we instead observe slowing of synaptic inhibitory kinetics, a reduction in synaptic inhibitory frequency, and a change in receptor subunit expression. Short-term synaptic depression of inhibitory currents was significantly reduced in prepubescent Fmr1-KO mPFC. Finally, we identify fast-spiking interneurons as partial mediators of reduced short-term inhibitory synaptic depression. Together, our data provide the first evidence of GABAergic pre- and post-synaptic changes in the mPFC of Fmr1-KO mice.

## Materials and Methods

### Animals

Only male mice were used for all experiments described in this work. Fmr1 knockout (KO; DutchBelgianFXSConsortium, 1994), and wildtype (WT) littermate controls were generated by crossing heterozygote Fmr1 C57BL/6J females with WT C57BL/6J males. The females used for breeding originated from backcrossing on the C57BL/6J line (Charles River) for at least 10 generations. Experiments were carried following the European Communities Directive of 24th of November 1986 (86/609/EEC) and with approval of the local animal care and use committee of the Vrije Universiteit. For all experiments, prepubescent animals were between the ages of postnatal (P) day 14 to P21, and adolescent animals were between P42–P49, except the adolescent spontaneous IPSC group that ranges between P36–P42.

### mPFC Synaptosomal Preparation and Protein Analysis

Following swift decapitation, brains were removed and placed on an ice-cold platform. Three frontal cortex slices were excised, and excess tissue surrounding the mPFC was cut away. The remaining tissue was first frozen in dry ice and subsequently stored at −80°C until further use. mPFC samples from three animals were pooled together for each age and genotype, resulting in eight samples per age per genotype. Synaptosomal fractions, enriched in pre- and postsynaptic proteins (Li et al., 2004), were isolated following sucrose gradient-assisted biochemical fractionation. In brief, samples were homogenized in ice-cold 0.32 M sucrose (5% of homogenate was collected as to total tissue lysate) and then centrifuged at 1,000× *g* for 10 min. The supernatant was loaded on top of a sucrose gradient consisting of 0.85 M and 1.2 M sucrose. After centrifugation at 100,000× *g* for 2 h, the synaptosomal fraction at the interface of 0.85 M/1.2 M sucrose was collected. Following the last centrifugation step (71,000× *g* for 30 min), the resulting pellet was dissolved in 70 μl 5 mM Hepes (pH 7.4) and stored at −80°C.

For each sample, protein concentration was determined with a Bradford assay (Bio-Rad Laboratories) and 5 μg protein was used for immunoblotting. Samples were lysed in Laemmli lysis buffer, separated by electrophoresis on gradient precast gels (4–20% Criterion TGX stain-free, Bio-Rad Laboratories), and blotted to PVDF membrane (Bio-Rad Laboratories). Samples were loaded in alternate order, so as each KO sample ran adjacent to a WT sample. The following primary antibodies were used: mouse anti-GABA_A_ α1 (1:1,000, Neuromab), rabbit anti-GABA_A_ α2 (1:500, Novus Biologicals), mouse anti-GABA_A_ β3 (1:500, Neuromab), rabbit anti-GABA_A_ γ2 (1:1, 000, Thermo Scientific -Pierce), mouse anti-GABA_B_ R1 (1:500, Neuromab), mouse anti-GABA_B_ R2 (1:1,000, Neuromab), rabbit anti-GAT1 (1:1,000, Chemicon/Millipore), goat anti-gephyrin (1:500, Santa Cruz). After incubation with horseradish peroxidase-conjugated secondary antibody (1:10,000 or 1:5,000; Dako, Glostrup) and visualization with Femto Chemiluminescent Substrate (Thermo Scientific) blots were scanned using the Li-Cor Odyssey Fc (Westburg) and analyzed with Image Studio (Li-Cor). Total protein was visualized using trichloro-ethanol staining, scanned using Gel Doc EZ imager (BioRad Laboratories) and analyzed with Image Lab (BioRad Laboratories) to correct for input differences per sample, as this is a reliable method not dependent on a single protein for normalization (Van den Oever et al., 2010). In brief, we corrected for input differences by taking the ratio between each band and the corresponding gel lane (Supplementary Figure S3). For quantification, intensity value was expressed as a ratio between that value and the WT group average, namely Sample1 = (Sample1)/(WT average), and so on. As such, the group average for WT ratios equaled to 1 (±SEM). Accordingly, fold change vs. WT was represented in the ratio of the KO group average. Differences between genotypes were assessed with either the Student’s *t*-test or with the Mann–Whitney *U* test. For both prepuberty and adolescence, one WT and one KO sample were excluded from statistical analysis as outliers (>2 × SD).

### Slicing

For spontaneous, miniature, evoked, and unitary recordings tissue was prepared as follows. Mice were swiftly decapitated and brains were extracted in ice-cold choline solution (110 mM choline chloride, 11.6 mM Na-ascorbate, 7 mM MgCl_2_, 3.1 mM Na-pyruvate, 2.5 mM KCl, 1.3 mM NaH_2_PO_4_, 0.5 mM CaCl_2_, 26 mM NaHCO_3_, 10 mM glucose, at ~300 mOsm, pH 7.4) continuously gassed with carbogen mixture (95% O_2_ and 5% CO_2_). Subsequently, the brain was mounted and acute coronal slices 300–350 μm were obtained using a vibrating microtome (Microm) while submerged in ice-cold choline solution, continuously gassed with carbogen. Slices were left to recover for <5 min in room temperature choline solution before being transferred into a slice chamber containing continuously carbogen gassed ACSF (125 mM NaCl, 3 mM KCl, 1.2 mM NaH_2_PO_4_, 1 mM MgSO_4_, 2 mM CaCl_2_, 26 mM NaHCO_3_, 10 mM glucose, ~ at 300 mOsm, pH 7.4) for at least 1 h before recordings.

### Electrophysiology

For spontaneous, miniature, evoked, and unitary recordings, measurements were conducted in the presence of AMPA receptor blocker CNQX (10 μM, Abcam), NMDA receptor blocker DL-AP5 (50 μM, Abcam), and the GABA_B_ receptor blocker CGP55845 (4 μM, Tocris). For miniature IPSC recordings, tetrodotoxin (1 μM, Abcam) was additionally used to block Na^+^ gating channels. Slices were transferred to a submerged recording chamber and left to equilibrate for 10 min under continuous perfusion of ~2 ml/min of ACSF solution at 32°C. mPFC was selected under visual guidance from differential interference contrast microscopy and layer V pyramidal cells were identified based on their distance from the midline, morphology, and responses to current injections of 1,000 ms from −200 pA to +100 pA at 25 pA steps. Whole-cell recordings were conducted using borosilicate glass pipettes (2.5–5.5 MΩ) containing high Cl^−^ intracellular solution (70 mM K-Gluconate, 70 mM KCl, 10 mM Hepes, 4 mM Mg-ATP, 4 mM K2-phosphocreatine, 0.4 mM GTP, 0.2% biocytin, at 280–290 mOsm, 7.2–7.3 pH). Cells were held at −70 mV and recordings were terminated if series resistance changed by more than 20% during recordings; cells were rejected if access resistance was greater than 20–25 MΩ. Recordings were acquired with pClamp software (Molecular Devices), using a Multiclamp 700B amplifier (Molecular Devices), low-pass filtered at 3 kHz, and digitized with an Axon Digidata 1440A (Molecular Devices). Sampling frequency for spontaneous and miniature IPSCs was at 10 kHz, and for evoked and unitary STP at 50 kHz.

### Spontaneous and Miniature IPSCs

For sIPSC and mIPSC recordings, a total of 15 min were recorded per cell, and only the last 10 min were analyzed, to allow for equilibration and stabilization of the patch. Data reported are from the following number of animals (a) and cells (c): Pre-puberty [sIPSC WT(4a, 12c) KO(4a, 12c), mIPSC WT(6a, 9c) KO(7a, 10c)], Adolescence [sIPSC WT(7a, 14c) KO(9a, 11c)], mIPSC WT(5a, 13c) KO(5a, 14c). Traces were analyzed with mini Analysis software (Synaptosoft) to extract frequency, amplitude, rise time (10%–90% of peak amplitude), decay kinetics, and current waveforms. Biexponential fittings of spontaneous, miniature, end evoked IPSC decays were also conducted in mini Analysis, on a subset of events that were evaluated for the goodness of such fits, from an original set of ~400 randomly selected events, using the following equation: *f*(t) = Ifast*exp(–t/taufast) + Islow*exp(–t/tauslow). Weighted tau was calculated using the following equation: τweighted = (τfast*Ifast + τslow*Islow) / (Ifast + Islow). General amplitude histograms at 3 pA bin widths were generated from a random selection of 5,000 events per genotype/pharmacology/age from the pool of all events per condition, to avoid confounding effects due to differences in total numbers. Non-parametric probability density fits were performed using the statistics toolbox of Matlab (Mathworks).

### Evoked Short-Term Plasticity (eSTP)

Data reported are from the following number of animals (a) and cells (c): Pre-puberty [WT(7a, 10c) KO(7a, 11c)], Adolescence [WT(7a, 8c) KO(6a, 8c)]. Upon achieving a stable whole-cell patch with pyramidal cells, a unipolar stimulating electrode was positioned at a distance of ≤100 μm from the soma. Test pulses were delivered to assess the location, and recordings proceeded if stable and uniform (<2 ms rise time 10%–90%) responses could be observed. Injection current magnitude was set to yield ≥ of half-maximal synaptic responses, ranging from 20 μA to 50 μA. The short-term plasticity protocol was initiated, by delivering five pulses at frequencies of either 5 Hz, 20 Hz, 50 Hz, or 100 Hz, with a recovery pulse delivered 500 ms after the 5th pulse. The stimulation regime cycled between these frequencies, with an inter-sweep interval of 16 s, until a total of 15–30 sweeps were recorded for each frequency. Sweep duration was set to 2 s and the pulse duration was set at 300 μs. A small voltage step was included 80 ms from the start of each sweep to monitor the stability of the recording. Current injections were mediated through a Master-9 pulse stimulator and an ISO-flex stimulus isolator (A.M.P.I). For both ages and genotypes, 1–2 cells per group exhibited an averaged potentiation of responses and were excluded from the analysis. A low number of failures were observed under our eSTP protocol, and such sweeps were removed during analysis, leaving 20–25 sweeps per frequency. In a few cells, especially at 100 Hz, the number of failures was increased and a lower number of sweeps were used, typically 10–15.

### Unitary Short-Term Plasticity (uSTP)/Short-Term Depression (STD)

Data reported are from the following number of animals (a) and cells (c): Pre-puberty [WT(4a, 4c(6c for 50 Hz) KO(7a, 11c)]. Upon achieving a stable whole-cell patch with pyramidal cells, fast-spiking interneurons in the vicinity were identified by the rounded morphology and patched. Action potential profiles of putative interneurons were generated and if they matched profiles of fast-spiking cells, current injections were initiated to probe for a unitary inhibitory connection between the interneuron and the pyramidal cell. If a unitary inhibitory to excitatory connection was present, the uSTP protocol was initiated, akin to the eSTP protocol. In uSTP however, each pulse was generated by supra-threshold current injections, for 2 ms at 1,800 pA per pulse, delivered *via* the patch pipette to the interneuron to elicit presynaptic action potentials. uSTP frequencies were prioritized, recorded as sets, and were not cycled. This was done to maximize complete data sets, and frequencies were collected in the order of 50 Hz, 20 Hz, 5 Hz, and 100 Hz. A maximum of 25 sweeps was averaged per frequency per cell, with a minimum of 10 sweeps for some cells at the highest frequency. Due to the high degree of failures for at least one of the five pulses, no sweeps were excluded based on that.

### Quantal Analysis and Peak-Scaled Non-stationary Noise (PSnSN) Analysis of mIPSCs

The quantal analysis relies on the hypothesis that the variation in post-synaptic responses occurs at multiples of the current elicited by elementary release (Bekkers, 1994). As such amplitude histograms can be described by multiple equidistant Gaussian distributions, reflecting discrete post-synaptic responses upon elementary release. For each cell events ranging from 5 pA to 150 pA amplitudes were binned at 2 pA increments, and best-fit functions were determined by the least-squares method along with visual confirmation of the fits (Edwards et al., 1990; Vislay et al., 2013).

In the absence of single-channel measurements, the use of PSnSN analysis on the decay current waveform can provide accurate estimates of single-channel currents, and the number of post-synaptic receptors open during peak current (De Koninck and Mody, 1994; Hartveit and Veruki, 2007). From each cell 50–500 events were selected, ensuring no overlapping events occurred during their decay phase. The mean response from all events per cell was calculated, scaled to the peak amplitude of each event, and subsequently subtracted from that event, generating a matrix of the remainders from each event per cell. The variance of this matrix was calculated across each index point. Next, the amplitude of the mean response of the cell was divided into 25 bins, and the time (index) ranges corresponding to each amplitude bin were used to calculate the mean-variance (σ^2^) from the remainders matrix, for each amplitude bin (I). The relationship between mean amplitude bin and corresponding variance was fitted with the following equation: σ^2^(I) = (i*I) - (I^2^/N) + σ_b_^2^, where (i) corresponds to the single-channel current, (N) corresponds to the number of receptors open during peak, and (σ_b_^2^) is mean-variance of baseline. Unitary conductance was calculated by dividing the single-unit currents with the driving force corresponding to the intracellular and extracellular Cl^−^ concentrations in our preparations.

### Tsodyks-Markram Phenomenological Synaptic Transmission Model

eSTP dynamics were further analyzed using the Tsodyks-Markram phenomenological synaptic transmission model as described before (Tsodyks and Markram, 1997; Loebel et al., 2009; Testa-Silva et al., 2012).

### Data Analysis and Statistics

Analyses of Quantal distributions, PSnSN, Tsodyks-Markram modeling, eSTP, and uSTP, were performed with custom-built Matlab (Mathworks) scripts. Fittings of eSTP decay current waveforms were performed in GraphPad (Prism), using the same functions as with spontaneous and miniature IPSCs. Statistical tests were performed as described in each figure legend and the Supplementary Table S1. Normality distribution was assessed with either the D’Agostino-Pearson omnibus normality test or the Shapiro-Wilk normality test. Welch’s correction was applied in cases where parametric data exhibited the unequal distribution of variances.

## Results

### Altered GABA_A,B_ Subunit Expression in Adolescent Fmr1-KO mPFC During

**Figure 1:**
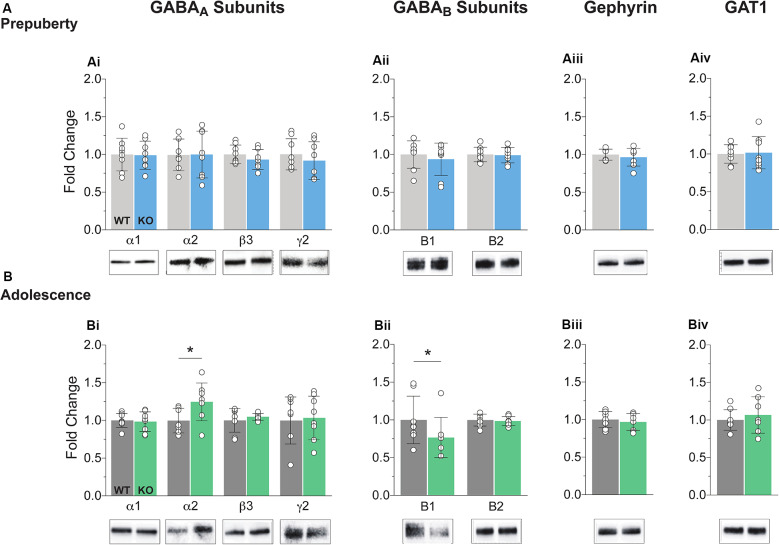
Protein expression profiles of GABA components in Fragile X Mental Retardation 1 (FMR1)-knockout (KO) medial prefrontal cortex (mPFC). Western blot analysis of selected GABA_A_ subunits **(Ai,Bi)**, the two GABA_B_ subtypes **(Aii,Bii)**, gephyrin **(Aiii,Biii)**, and GABA transporter 1 **(Aiv,Biv)**, in mPFC, enriched synaptosomal fractions from Fmr1-KO and wildtype (WT) age-matched littermates. **(A)** No difference in protein expression in any of the GABA machinery components tested was found in prepubescent Fmr1-KO mPFC. **(B)** GABA_A_ receptor subunit α2 expression was enhanced during adolescence (**Bi**, WT_(*n* = 7)_ = 0.93 ± 0.16, KO_(*n* = 8)_ = 1.18 ± 0.25, *p* = 0.04), while expression of GABA_B_ receptor subtype B1 was found reduced during the same postnatal developmental period (**Bii**, WT_(*n* = 8)_ = 0.99 ± 0.31, KO_(*n* = 8)_ = 0.77 ± 0.27, *p* = 0.03). Depending on the sample distributions, differences between genotypes were assessed with either the Student’s *t*-test or the Mann–Whitney *U* test. For all panels, asterisks denote statistical significance of *p < 0.05. Error bars represent SD.

The precise composition of GABAergic receptors and the degree of expression of GABAergic auxiliary proteins is both a cause and a therapeutic target for several neuropathological conditions including FXS (Fritschy et al., 1994; Lozano et al., 2014). Synaptosomal fractions were prepared from mPFC enriched protein lysates (see “Materials and Methods” section) and analyzed for the expression of several GABA subunits and auxiliary proteins (Figure 1, Supplementary Figure S3). No differences in protein expression for either GABA_A_ or GABA_B_ components tested were reported during prepubescence (Figures 1Ai,ii). However, during adolescence, an expression of GABA_A_ receptor α2 subunit was increased in the Fmr1-KO mPFC (Figure 1Bi, *p* = 0.04). The expression of the GABA_B_ receptor B1 subtype was found reduced compared to WT littermate controls (Figure 1Bii, *p* = 0.03). During either postnatal developmental times no difference in the expression of gephyrin (Figures 1Aiiii,Biii), or GABA transporter 1 (GAT1, Figures 1Aiv,Biv) was observed. An increase in α2 GABA_A_ subunit and a reduction in GABA_B_ receptor subtype B1 expression is suggestive of dysregulated inhibitory signaling in adolescent FXS mPFC.

### Action Potential Dependent IPSC Frequency Changes in Fmr1-KO mPFC

**Figure 2:**
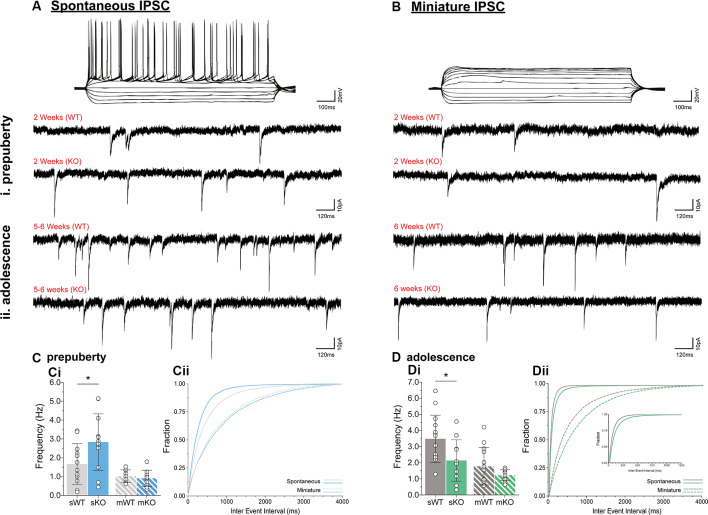
Prepubescent and adolescent mPFC inhibitory postsynaptic currents. Example traces of action potential profiles and whole-cell spontaneous (no tetrodotoxin, **A**) and miniature (1 μM tetrodotoxin, **B**) IPSCs from layer V mPFC pyramidal cells in prepubescent **(Ai,Bi)** and adolescent **(Aii,Bii)** Fmr1-KO and WT mPFC. Recordings were conducted in the continuous presence of AMPA, NMDA, and GABA_B_ receptor blockers (see “Materials and Methods” section). **(C)** The frequency of prepubescent sIPSCs was increased (**Ci**, WT_(*n* = 12)_ = 1.68 ± 1.09 Hz, KO_(*n* = 12)_ = 2.84 ± 1.50 Hz, *p* = 0.04), and shorter inter-event intervals were observed (solid blue line, **Cii**). In the presence of tetrodotoxin no difference was found in prepubescent IPSC frequency (**Ci**, WT_(*n* = 9)_ = 1.03 ± 0.34 Hz, KO_(*n* = 11)_ = 0.91 ± 0.44 Hz), or in inter-event interval times (broken lines, **Cii**). **(D)** Adolescent IPSC frequency was significantly reduced in spontaneous (**Di**, WT_(*n* = 14)_ = 3.49 ± 1.46 Hz, KO_(*n* = 11)_ = 2.14 ± 1.28 Hz, *p* = 0.03) but not miniature recordings (**Di**, WT_(*n* = 13)_ = 1.78 ± 1.16 Hz, KO_(*n* = 14)_ = 1.23 ± 0.34 Hz, *p* = 0.12). Inter-event interval times for both genotypes were overall shorter as expected developmentally, with an observable shift towards longer times in spontaneous (solid green lines, **Dii**) and miniature (broken green lines, **Dii**) IPSCs in Fmr1-KO recordings. Genotypic differences were assessed with the Student’s *t*-test. For all panels, asterisks denote statistical significance of *p < 0.05. Error bars represent SD.

To assess putative changes in functional inhibition onto layer V mPFC pyramidal neurons, inhibitory postsynaptic currents (IPSCs) were studied in the absence (spontaneous IPSC, Figure 2A) or presence (miniature IPSC, Figure 2B) of Na^+^ channel blocker tetrodotoxin, during prepuberty (Figure 2C) and adolescence (Figure 2D). Fmr1-KO sIPSC frequency was increased during prepubescence (Figure 2Ci, *p* = 0.04) with a shift toward shorter inter-event intervals (solid blue line, Figure 2Cii). During adolescence, however, Fmr1-KO sIPSC frequency was significantly reduced compared to WT littermate controls (Figure 2Di, *p* = 0.03). These changes were not observed in mIPSC recordings during either prepubescence (Figure 2Ci, *p* = 0.53) or adolescence (Figure 2Di, *p* = 0.12). Together with our data reveal dynamic and activity-dependent changes of prepubescent and adolescent GABA_A_ mediated inhibitory drive in Fmr1-KO mPFC.

### Prolonged GABA_A_ Receptor Kinetics in Adolescent Fmr1-KO mPFC

**Figure 3:**
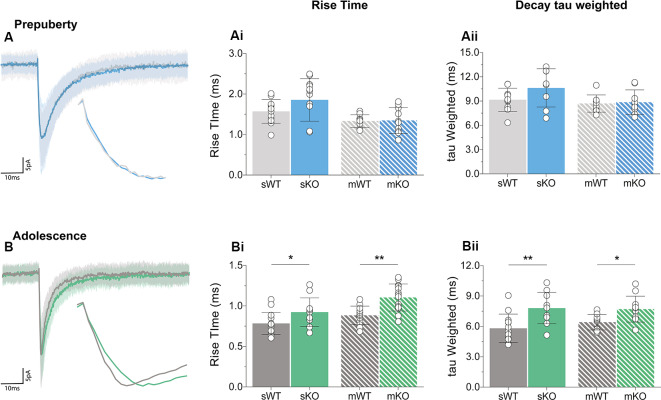
Receptor activation and decay times significantly prolonged in adolescent mPFC. Averages of 50–100 example IPSCs (Average: solid lines, SD: shaded area) during pre-puberty **(A)** and adolescence **(B)**. During pre-puberty both synaptic rise times [**Ai**, sIPSC (WT_(*n* = 12)_ = 1.57 ± 0.29 ms, KO_(*n* = 12)_ = 1.86 ± 0.53 ms, *p* = 0.12), mIPSC *p* = 0.89] and weighted tau of decays [**Aii**, sIPSC (WT_(*n* = 10)_ = 9.15 ± 1.43 ms, KO_(*n* = 8)_ = 10.63 ± 2.37 ms, *p* = 0.12), mIPSC *p* = 0.80] were similar for the two genotypes in spontaneous and miniature IPSC recordings. **(B)** Receptor activation times were significantly prolonged during adolescence in both conditions [**Bi**, sIPSC (WT_(*n* = 14)_ = 0.78 ± 0.14 ms, KO_(*n* = 11)_ = 0.92 ± 0.18 ms, *p* = 0.04), mIPSC (WT_(*n* = 13)_ = 0.88 ± 0.11 ms, KO_(*n* = 14)_ = 1.10 ± 0.17 ms, *p* < 0.01)]. Additionally, adolescent weighted tau of decay was also prolonged [**Bii**, sIPSC (WT_(*n* = 13)_ = 5.81 ± 1.41 ms, KO_(*n* = 10)_ = 7.81 ± 1.56 ms, *p* < 0.01), mIPSC (WT_(*n* = 10)_ = 6.41 ± 0.76 ms, KO_(*n* = 11)_ = 7.70 ± 1.28 ms, *p* = 0.01)], independently of tetrodotoxin application (Supplementary Table S1). Genotypic differences were assessed with the Student’s *t*-test. For all panels asterisks denote statistical significances of **p < 0.01, *p < 0.05. Error bars represent SD.

Receptor kinetics orchestrate precise transitions between open and close channel states, and changes in stoichiometry, neurotransmitter levels, or general synaptic morphology, can impact gating currents. Prepubescent GABA_A_ synaptic rise time and weighted tau (tauW) of decay were equal between genotypes (Figure 3A, Supplementary Figure S2, Rise Time *p* = 0.12, tauW *p* = 0.12). By adolescence, Fmr1-KO synaptic kinetics slowed down substantially, exhibiting prolonged synaptic activation times (Figure 3Bi, sIPSC *p* = 0.04, mIPSC *p* < 0.01) and tauW of decay (Figure 3Bii, sIPSC *p* < 0.01, mIPSC *p* = 0.01), in an action potential independent manner (Supplementary Table S1). This slowdown coincides with the increase in α2 subunit expression, shown to prolong receptor decays (Lavoie et al., 1997), promoting extended charge transfer times.

### Enhanced IPSC Amplitudes in Prepubescent Fmr1-KO mPFC

**Figure 4:**
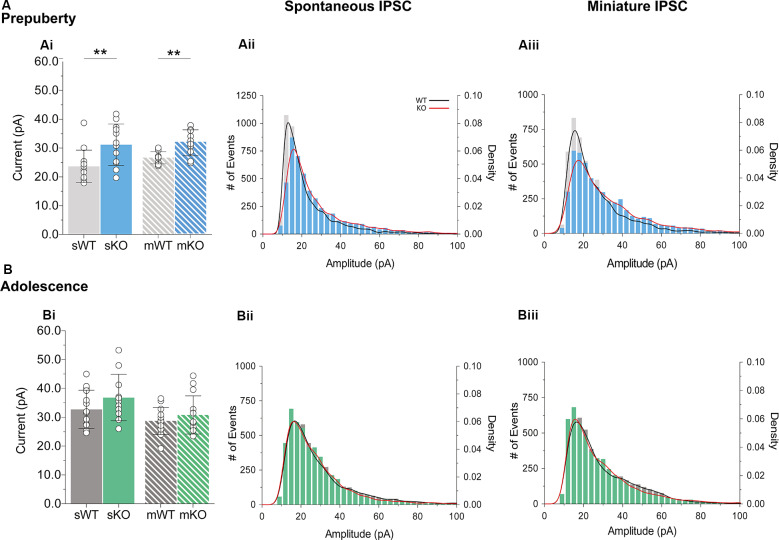
IPSC amplitude potentiation in prepubescent Fmr1-KO mPFC. **(A)** Prepubescent IPSC amplitudes were found potentiated regardless of Na^+^ channel blockade [**Ai**, sIPSC (WT_(*n* = 12)_ = 23.64 ± 5.62 pA, KO_(*n* = 12)_ = 31.18 ± 7.17 pA, *p* < 0.01), mIPSC (WT_(*n* = 9)_ = 26.65 ± 2.12 pA, KO_(*n* = 10)_ = 31.83 ± 4.50 pA, *p* < 0.01)]. **(B)** During adolescence sIPSC [**Bi**, (WT_(*n* = 14)_ = 32.75 ± 6.64 pA, KO_(*n* = 11)_ = 36.82 ± 8.05 pA, *p* = 0.18)] and mIPSC [**Bi**, (WT_(*n* = 13)_ = 28.72 ± 4.72 pA, KO_(*n* = 14)_ = 30.79 ± 6.64 pA, *p* = 0.36)] amplitudes were equal between genotypes. Histograms of amplitude distributions for sIPSC **(Aii, Bii)** and mIPSC **(Aiii,Biii)** revealed a drop at small amplitudes along with a shift toward larger amplitude bins during pre-puberty **(Aii,Aiii)**, whereas no observable shift in amplitude distributions was observed during adolescence **(Bii,Biiii)**. Solid lines represent WT (black) and Fmr1-KO (red) probability density plots of the underlying amplitude distributions. Genotypic differences were assessed with the Student’s *t*-test. For all panels asterisks denote statistical significances of **p < 0.01. Error bars represent SD.

**Figure 5:**
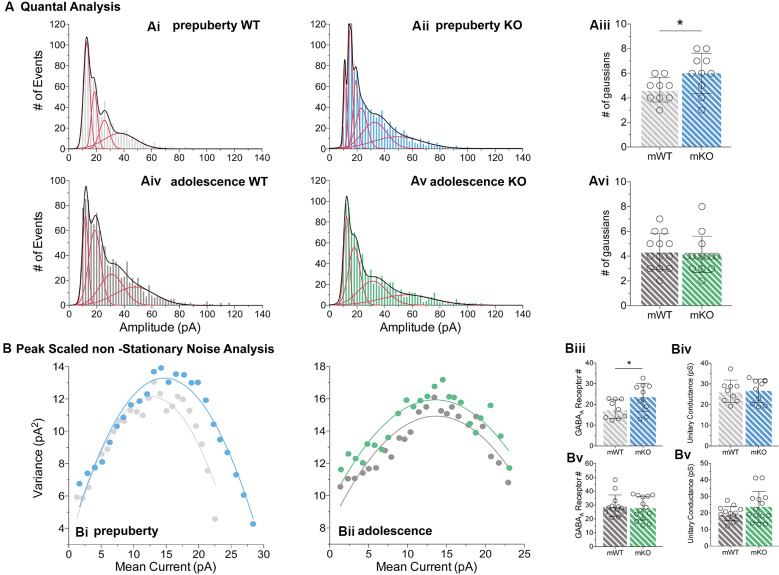
Quantal and peak scaled non-stationary noise analysis of miniature prepubescent and adolescent IPSCs. Quantal **(A)** and peak scaled non-stationary **(B)** analyses were conducted on mIPSC recordings to dissect putative synaptic changes driving increases in amplitude. **(A)** Example analysis of quantal distributions from individual cells during pre-puberty **(Ai,Aii)** and adolescence **(Aiv,Av)**. Amplitudes from each cell were binned at 2 pA increments, and the number of individual Gaussians (red lines) that best fit the overall distributions (black line, a sum of Gaussians) was assessed. During pre-puberty a larger number of Gaussians best inscribed the amplitude distributions of Fmr1-KO mIPSCs (**Aiii**, WT_(*n* = 9)_ = 4.67 ± 1.00 #fits, KO_(*n* = 10)_ = 6.00 ± 1.63 #fits, *p* < 0.05), whereas during adolescence an equal number of distributions described the data between the genotypes (**Avi**, WT_(*n* = 13)_ = 4.39 ± 1.45 #fits, KO_(*n* = 14)_ = 4.14 ± 1.46 #fits, *p* = 0.67). **(B)** Example plots from prepubescent **(Bi)** and adolescent **(Bii)** Peak-Scaled non-Stationary Noise (PSnSN) analysis of noise fluctuations around the mean of the decay phase of current waveforms in mIPSCs. The number of receptors open during peak current was found increased in prepubescent Fmr1-KO recordings (**Biii**, WT_(*n* = 9)_ = 17.75 ± 4.46 #Rs, KO_(*n* = 10)_ = 23.47 ± 6.71 #Rs, *p* < 0.05), and this difference was normalised by adolescence (**Bv**, WT_(*n* = 13)_ = 29.59 ± 7.74 #Rs, KO_(*n* = 14)_ = 27.70 ± 8.35 #Rs, *p* = 0.55). No difference in unitary conductance was observed during either postnatal developmental time point: Pre-puberty (**Biv**, WT_(*n* = 9)_ = 26.44 ± 5.42 pS, KO_(*n* = 10)_ = 26.66 ± 5.67 pS, *p* = 0.94), Adolescence (**Bvi**, WT_(*n* = 13)_ = 19.62 ± 4.25 pS, KO_(*n* = 14)_ = 23.66 ± 9.28 pS, *p* = 0.16). Genotypic differences were assessed with the Student’s *t*-test. For all panels, asterisks denote statistical significance of *p < 0.05. Error bars represent SD.

During prepubescence, synaptic amplitudes in Fmr1-KO were larger for both spontaneous and miniature IPSCs (Figure 4Ai, sIPSC *p* < 0.01, mIPSC *p* < 0.01). We did not observe any differences in input resistance (WT vs. KO: sIPSC 122.49 ± 36.91 MΩ vs. 140.08 ± 48.62 MΩ, *p* = 0.35; mIPSC 170.05 ± 48.73 MΩ vs. 179.45 ± 60.83 MΩ, *p* = 0.74, data not shown), or capacitance (WT vs. KO: sIPSC 208.75 ± 59.49 pF vs. 185.54 ± 30.25 pF, *p* = 0.29; mIPSC 161.73 ± 23.13 pF vs. 149.95 ± 25.96 pF, *p* = 0.39, data not shown) between these cells. Prepubescent amplitude distributions from Fmr1-KO cells exhibited a reduction in smaller currents with a parallel shift towards larger amplitude bins in both recording conditions (Figures 4Aii,iii). During adolescence, sIPSC and mIPSC amplitudes were comparable between the two genotypes (Figure 4Bi, sIPSC *p* = 0.18, mIPSC *p* = 0.36), and no differences in amplitude distributions were observed (Figures 4Bii,iii). The nature of synaptic current amplitude distributions can reveal the number of discrete post-synaptic responses underlying the variations in amplitudes, deduced by the number of Gaussians best fitting amplitude distributions (Edwards et al., 1990). To that end, we performed quantal analysis on mIPSC amplitudes (Figure 5A), during prepuberty (Figures 5Ai,ii) and adolescence (Figures 5Aiv,v). In line with the increased IPSC amplitudes during prepubescence, a greater number of distributions better fitted Fmr1-KO amplitudes as compared to WT littermates (Figure 5Aiii, *p* < 0.05). During adolescence, no difference in the number of Gaussians that best fitted the data was observed (Figure 5Avi, *p* = 0.67). Our analysis of IPSCs received by layer V mPFC pyramidal cells demonstrates an early potentiation in amplitudes along with an expansion in discrete post-synaptic responses that normalizes during adolescence.

To better define the nature of the post-synaptic changes observed, we applied PSnSN analysis on synaptic current waveforms (Figure 5B), during prepuberty (Figure 5Bi) and adolescence (Figure 5Bii). Such analysis can reveal the single-channel current and the average number of receptors open at peak (Hartveit and Veruki, 2007). During prepubescence, PSnSN analysis revealed a significantly greater number of receptors open during peak activation in Fmr1-KO cells (Figure 5Biii, *p* < 0.05), without a change in unitary conductance (Figure 5Biv, *p* = 0.94). During adolescence, the number of receptors open at peak current (Figure 5Bv, *p* = 0.55) and unitary conductance (Figure 5Bvi, *p* = 0.16) was similar between genotypes. Therefore, the observed action potential independent shift towards larger prepubescent amplitudes can partly be due to increased receptor numbers open during peak activation. Whether this reflects a general increase in total receptor numbers per synapse or an increase in the number of receptors engaged, can not be deduced from this analysis. Nevertheless, heightened prepubescent GABA receptor activation leads to stronger post-synaptic inhibition onto layer V mPFC pyramidal cells.

### Reduced Short-Term Inhibitory Depression in Prepubescent Fmr1-KO mPFC

**Figure 6:**
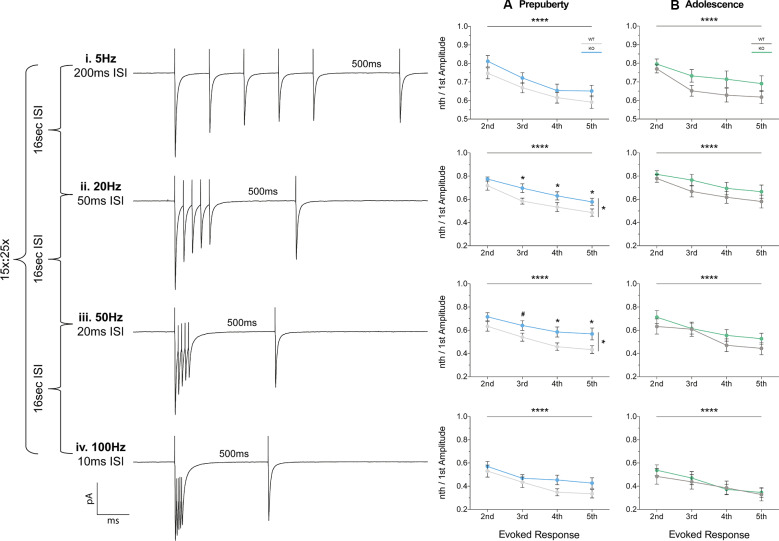
Short term plasticity of evoked IPSC is reduced in prepubescent Fmr1-KO mPFC. Average responses from a single data set at each frequency tested **(i–iv)**. **(A)** During pre-puberty a reduction in short-term inhibitory depression was observed at 20 Hz [**Aii**, Supplementary Table S1, Evoked Response: 2nd (WT_(*n* = 10)_ = 0.72 ± 0.04, KO_(*n* = 11)_ = 0.77 ± 0.02), 3rd (WT_(*n* = 10)_ = 0.58 ± 0.04, KO_(*n* = 11)_ = 0.70 ± 0.04), 4th (WT_(*n* = 10)_ = 0.53 ± 0.04, KO_(*n* = 11)_ = 0.63 ± 0.04), 5th (WT_(*n* = 10)_ = 0.49 ± 0.03, KO_(*n* = 11)_ = 0.58 ± 0.03)], and 50 Hz stimulations [**Aiii**, Supplementary Table S1, Evoked Response: 2nd (WT_(*n* = 10)_ = 0.63 ± 0.04, KO_(*n* = 11)_ = 0.72 ± 0.04), 3rd (WT_(*n* = 10)_ = 0.54 ± 0.04, KO_(*n* = 11)_ = 0.64 ± 0.04), 4th (WT_(*n* = 10)_ = 0.49 ± 0.03, KO_(*n* = 11)_ = 0.59 ± 0.04), 5th (WT_(*n* = 10)_ = 0.43 ± 0.03, KO_(*n* = 11)_ = 0.57 ± 0.05)], while for 5 Hz **(Ai)** and 100 Hz **(Aiv)** responses were comparable between the two genotypes. **(B)** By adolescence no differences in the amount of depression were observable at all frequencies tested (**Bi–iv**, Supplementary Table S1). Differences were assessed with two-way-Repeated Measures ANOVA (Supplementary Table S1). For all panels, asterisks denote statistical significance of ****p < 0.0001, *p ≤ 0.05, and ^#^corresponds to 0.05 < *p* ≤ 0.1. Error bars represent SEM.

Short-term plasticity (STP) reflects use-dependent dynamic changes in synaptic transmission during repeated presynaptic activity, and it is believed to participate in neuronal information processing (Zucker and Regehr, 2002). To study STP a stimulating electrode was used to deliver five pulses at frequencies of 5 Hz, 20 Hz, 50 Hz, and 100 Hz, and a recovery pulse was evoked at 500 ms after the 5th response (Figures 6i–iv). This protocol resulted in progressively depressing plasticity. No differences in the magnitude of the first response were observed between the two genotypes, during either prepubescence or adolescence over all frequencies (Supplementary Figures 1Ai–Bi, Supplementary Table S1). During prepubescence stimulation at intermediate frequencies resulted in reduced inhibitory synaptic depression in Fmr1-KO, both at 20 Hz [Figure 6Aii, Supplementary Table S1, two-way-RM-ANOVA; Genotype (*F*_(1,19)_ = 4.49, *p* = 0.04), Evoked Response (*F*_(3,57)_ = 50.14, *p* < 0.0001), Interaction (*F*_(3,57)_ = 0.82, *p* = 0.49), Subjects (*F*_(19,57)_ = 9.7, *p* < 0.0001)] and at 50 Hz [Figure 6Aiii, Supplementary Table S1, two-way-RM-ANOVA; Genotype (*F*_(1,19)_ = 4.75, *p* = 0.04), Evoked Response (*F*_(3,57)_ = 29.37, *p* < 0.0001), Interaction (*F*_(3,57)_ = 0.72, *p* = 0.55), Subjects (*F*_(19,57)_ = 12.38, *p* = 0.0001)]. By adolescence, the degree of inhibitory depression was comparable between genotypes (Figures 6Bii,iii, Supplementary Table S1). Therefore, prepubescent mPFC inhibitory STP was reduced, however, this reduction returned to WT levels by adolescence.

### Fast-Spiking Interneurons Partially Mediate Reduced Inhibitory Depression

**Figure 7:**
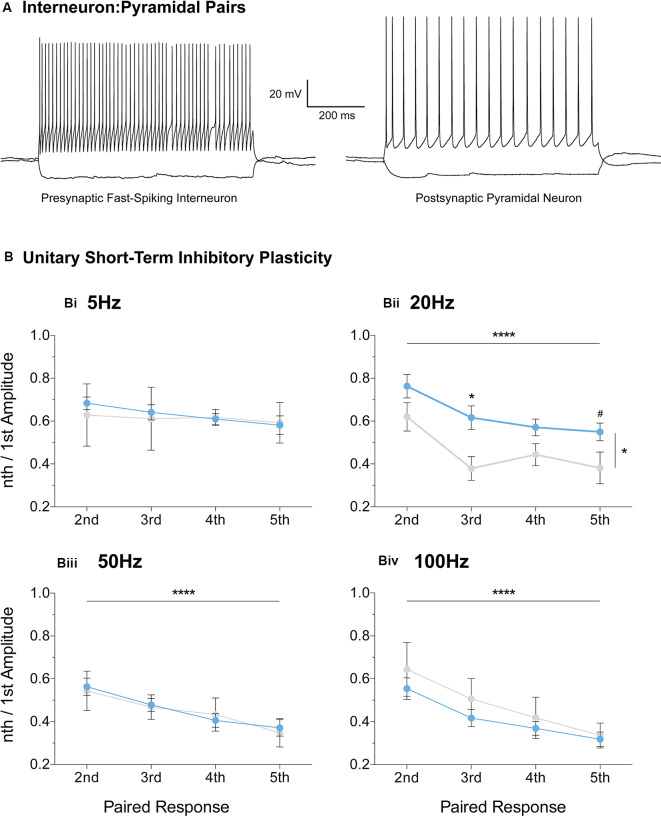
Reduced short-term inhibitory depression partially recapitulated in paired recordings of fast-spiking and pyramidal pairs. Fast-spiking interneurons in the vicinity of layer V pyramidal cells were patched **(A)** and unitary connectivity was probed *via* a stimulation protocol similar to the one applied for evoked STP (see “Materials and Methods” section). A reduction in unitary Short-Term Depression (STD) was observed in the Fmr1-KO mPFC at the 20 Hz stimulation frequency [**Bii**, Supplementary Table S1, Evoked Response: 2nd (WT_(*n* = 11)_ = 0.77 ± 0.06, KO_(*n* = 4)_ = 0.62 ± 0.07), 3rd (WT_(*n* = 11)_ = 0.61 ± 0.06, KO_(*n* = 4)_ = 0.38 ± 0.06), 4th (WT_(*n* = 11)_ = 0.55 ± 0.04, KO_(*n* = 4)_ = 0.44 ± 0.05), 5th (WT_(*n* = 11)_ = 0.56 ± 0.04, KO_(*n* = 4)_ = 0.38 ± 0.07)]. No difference in unitary inhibitory plasticity was observed at all other frequencies tested (Supplementary Table S1, **Bi,Biii,Biv**). Differences were assessed with two-way-Repeated Measures ANOVA (Supplementary Table S1). For all panels, asterisks denote statistical significance of ****p < 0.0001, *p < 0.05, and ^#^corresponds to 0.05 < *p* ≤ 0.10. Error bars represent SEM.

Furthermore, we extended the STP protocol, now between connected pairs of layer V mPFC pyramidal and fast-spiking interneurons (Figure 7). Unitary connections were subjected to a stimulation regime similar to the evoked IPSC protocol (Figures 6i–iv, see “Materials and Methods” section). No differences in unitary STP depression were observed between the two genotypes at 5 Hz, 50 Hz, and 100 Hz (Figures 7Bi,iii,iv, Supplementary Table S1). However, a significant attenuation in the depression of unitary STP responses was observed in Fmr1-KO connected pairs at 20 Hz [Figure 7Bii, Supplementary Table S1, two-way-RM-ANOVA; Genotype (*F*_(1,13)_ = 5.00, *p* = 0.04), Unitary Response (*F*_(3,39)_ = 9.71, *p* < 0.0001), Interaction (*F*_(3,39)_ = 0.58, *p* = 0.63), Subjects (*F*_(13,39)_ = 4.77, *p* = 0.0001)], akin to the one observed during the evoked STP protocol (Figure 6Aii). Our STP experiments reveal an activity-induced reduction in inhibitory synaptic depression, partially recapitulated in fast-spiking to pyramidal synaptic communication. Collectively, our data highlight dynamic GABAergic alternations at the molecular and functional levels, for the first time in the mFPC of FXS mice, during two crucial timepoints for postnatal prefrontal development.

## Discussion

GABAergic deficits in FXS are described in several brain areas in Fmr1-KO mice, and FXS patients suffer from increased susceptibility to seizures (Musumeci et al., 1999). We now extend the reach of inhibitory deficits to the mPFC, shown to underlie high order cognitive functions. Our data suggest sustained and dynamic inhibitory deficits during postnatal mPFC maturation. In prepubescent Fmr1-KO mPFC, IPSC frequency and amplitudes were increased while inhibitory synaptic depression was reduced. During adolescence inhibitory frequency was instead reduced, synaptic kinetics were prolonged, and expression of GABA receptor subunits deviated from WT. These data further support the impact FMRP imposes on GABAergic signaling both at the pre- and post-synaptic domains of inhibitory synapses. Importantly, continued inhibitory network imbalance during ongoing prefrontal circuit development and maturation, can severely hinder prefrontal mediated cognition, as seen in FXS.

### Enhanced Prefrontal Inhibition in Prepubescent Fmr1-KOs

Collectively, we observed enhanced inhibition during prepubescent prefrontal development in Fmr1-KOs. Increased inhibitory frequency and amplitudes, together with reduced inhibitory depression, promote stronger inhibition, possibly to counteract ongoing excitatory hyperconnectivity (Testa-Silva et al., 2012). Although the enhanced inhibition could restore network balance, it highlights significant inhibitory imbalances occurring in immature prefrontal circuits, that could fail to correctly establish and mature, thus negatively affecting prefrontal mediated cognition.

#### Increased Inhibitory Transmission

Fmr1-KO prepubescent IPSC frequency was increased in an action potential-dependent manner. Gephyrin expression was normal and no changes in mIPSC frequency were observed, suggesting that the number of inhibitory synapses was comparable between genotypes. Instead the frequency increase in sIPSCs may be due to a hyperconnected excitatory network that promotes elevated inhibitory drive. Indeed layer V excitatory cells in prepubescent Fmr1-KO mPFC are interconnected at higher probabilities than in WT (Testa-Silva et al., 2012). Notably, Fmr1-KO frequency returned to control levels upon action potential generation blockade (Supplementary Table S1). It is therefore proposed that the activity-dependent increase in IPSC frequency, in the absence of a change in the number of inhibitory synapses, is partially promoted by ongoing elevated levels of presynaptic excitatory drive.

#### Increased Post-synaptic Inhibitory Receptor Activation

Prepubescent Fmr1-KO IPSCs exhibited an action-potential independent increase of amplitudes. Changes in GABA levels can influence the current gated by receptors. Although general cortical GABA levels are found reduced in two-week-old Fmr1-KOs (Davidovic et al., 2011), neurotransmitter levels in juvenile and adult KO mPFC and frontal cortex are overall normal (Gruss and Braun, 2001, 2004). Furthermore, both absolute synaptic efficay—a measure of total synaptic resource utilization (Supplementary Figure S1, Tsodyks and Markram, 1997), and GAT1 expression levels—a measure of neurotransmitter reuptake rate (Scimemi, 2014), were equal between genotypes. Together, these findings suggest normal pre-synaptic GABA neurotransmitter levels and clearance in Fmr1-KO mPFC.

Notably, prepubescent Fmr1-KO mPFC excitatory cells are hyperconnected (Testa-Silva et al., 2012). Our prepubescent mIPSC quantal analysis, revealed an expanded number of discrete post-synaptic responses in Fmr1-KOs, suggesting increased inhibitory receptor activation in FXS. Homeostatic adaptations of inhibitory receptor numbers in response to dysregulated excitation, are an efficient way to restore network function (Otis et al., 1994; Kilman et al., 2002). Therefore, enhanced prefrontal inhibitory receptor activation in prepubescent Fmr1-KOs might be in response to the underlying hyperconnected excitatory network.

#### Reduced Inhibitory Short-Term Depression

STP represents use-dependent dynamic changes in synaptic transmission during repeated upstream stimulation, thereby integrating on-going synaptic activity essential to proper information processing (Zucker and Regehr, 2002; Regehr, 2012). Prepubescent Fmr1-KO inhibitory current depression was reduced for each subsequent event, most prominently at 20 Hz and 50 Hz frequencies. Ca^2+^ fluxes in synaptic compartments are central to STP dynamics (Zucker and Regehr, 2002). In line with this, L-type Ca^2+^ channel frontal levels are reduced in prepubescent Fmr1-KOs, and superficial mPFC Ca^2+^ signaling is shown to be unreliable (Chen et al., 2003; Meredith et al., 2007). Unreliable Ca^2+^ signaling can reduce release probability that in turn can attenuate synaptic depression upon repetitive stimulation. We did observe a reduction in prepubescent Fmr1-KO release probability (Supplementary Figure S1, *p* = 0.058). Yet this difference was just above significance raising the possibility that auxiliary mechanisms might also be at play. Post-synaptic receptor activation changes, akin to those discussed above, could also account for these reductions in inhibitory STD. Finally, this reduction in GABAergic STD can lead to changes in the excitability of prefrontal networks, corrupting proper information flow, and could underlie cognitive deficits in FXS models (Krueger et al., 2011; Kramvis et al., 2013).

Our data directly implicate fast-spiking interneurons (FS-INs) in the plasticity impairments we observe, as paired recordings with prepubescent Fmr1-KO mPFC pyramidal neurons also exhibited reduced inhibitory STD at 20 Hz. Interestingly, the L-type Ca^2+^ channels reduced in Fmr1-KO frontal cortex, also mediate FS-IN to pyramidal STP (Jensen and Mody, 2001). FS-INs are central to brain oscillations (Bartos and Elgueta, 2012), and the changes we observed in STP fall within frequency bands essential to proper cognitive processing and function (Schnitzler and Gross, 2005). Specifically, beta range oscillations—that include 20 Hz, enable the PFC to relay information to connected brain areas, including the hippocampus, amygdala, and visual cortices (Benchenane et al., 2011; Bygrave et al., 2019; Dal Monte et al., 2020). Thus beta oscillations afford the PFC to exert control over working-memory, social-decision making, and attention—cognitive domains affected in FXS. Notably, PFC beta-band long-range functional connectivity is reduced in FXS patients (van der Molen et al., 2014; Wang et al., 2017). Our work provides evidence of PFC FS-IN dysfunction that negatively impacts beta oscillations, providing thus a cellular target and temporal window of possible therapeutic intervention.

### Reduced Inhibitory Drive and Kinetics in Adolescent Fmr1-KOs

In contrast to enhanced prepubescent Fmr1-KO inhibition, during adolescence prefrontal inhibitory drive was reduced, and receptor kinetics slowed down. Although this could be a rebound response due to stronger prepubescent inhibition, it highlights further derailment of prefrontal inhibitory circuitry in FXS. As prefrontal cortices take longer to mature, a continued inhibitory network imbalance during ongoing circuit development and maturation can severely hinder prefrontal mediated cognition. Importantly, during adolescence the subunit expression changes we describe, can aid in devising more meaningful therapeutic strategies.

#### Reduced Inhibitory Frequency and Increased α_2_ Subunit Expression

During adolescence, the Fmr1-KO levels of receptors activated, IPSC amplitude, and STD returned to WT levels. Instead, the prefrontal inhibitory frequency was reduced and was activity-dependent, while inhibitory receptor kinetics were significantly slower in Fmr1-KOs. Decreases in PV^+^ cells have been reported in the mPFC of autistic patients (Hashemi et al., 2017), and Fmr1-KO somatosensory cortex (Selby et al., 2007), have also been observed in mPFC by us (Supplementary Figure S4) and can underlie the decrease in IPSC frequency. Furthermore, adolescent Fmr1-KO receptor kinetics were significantly prolonged compared to WT, while GABA_A_ subunit α_2_ expression was increased. Inhibitory receptors containing α_2_ subunits exhibit slower kinetics than those containing α_1_ (Lavoie et al., 1997: Dixon et al., 2014), and can thus compensate for reduced inhibitory frequency by extending the inhibitory charge transfer time. Also, augmentation of α_2, 3_-subunit activity rescues aberrant behavior in an autism model with reduced inhibitory frequency (Han et al., 2014). It is thus tempting to speculate that the increased α_2_ expression we observe could also be restorative, by counteracting reduced inhibitory drive through extending charge transfer times.

#### Reduced GABA_B_ B1 Expression

Furthermore, during adolescence, Fmr1-KO mPFC expression of the GABA_B_ B1 subtype was reduced. GABA_B_ B1 contains the pocket for GABA ligand binding, which also binds to baclofen—a pharmaceutical target in FXS clinical trials (Berry-Kravis et al., 2012, 2017; Frangaj and Fan, 2018). Although arbaclofen was well tolerated, it did not meet the primary clinical outcome of improved social avoidance in FXS (Berry-Kravis et al., 2017). If our observations are also reflected in FXS patients, then the prefrontal reduction in baclofen binding sites could render it a less effective pharmaceutical target, especially for prefrontal mediated cognitive domains. Compounds enhancing B2 subunit activity, alone or in combination with arbaclofen, or further augmenting inhibition *via* GABA_A_ a2 agonists, could increase outcome success.

## Conclusion

**Figure 8:**
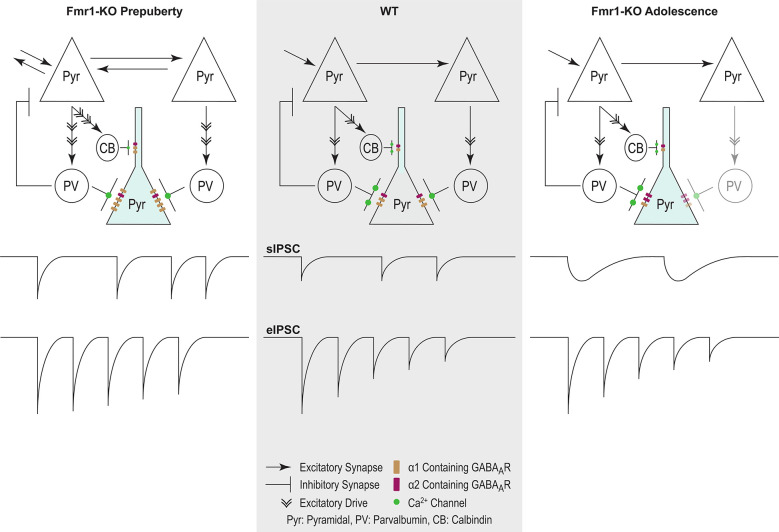
Proposed model of inhibitory changes in Fmr1-KO mPFC. Generic WT mPFC neuronal network. The model does not incorporate neurotypical developmental changes in inhibitory signaling. As such comparisons are between Fmr1-KO Pre-puberty and WT, and separately between Fmr1-KO Adolescence and WT. Fmr1-KO Prepubescence: compared to WT, the rate of sIPSC is increased in prepubescent Fmr1-KO mPFC. Enhanced excitatory drive due to hyper-connectivity between mPFC pyramidal cells is proposed to underlie the activity-dependent increase in sIPSC frequency. A putative increase in the number of post-synaptic GABA_A_Rs, as deduced from noise analysis, could promote the observed activity independent potentiation of sIPSC amplitudes. Finally, reduced probability of presynaptic release, possibly driven by attenuated expression of Ca^2+^ channels, can underlie the reduction in Fmr1-KO eIPSC depression. Fmr1-KO Adolescence: an activity independent slowdown of sIPSC kinetics was observed in adolescent Fmr1-KO mPSC compared to WT age-matched controls. Enhanced expression of α2 GABA_A_ subunit, if functionally incorporated, could partially explain receptor kinetic slowdown. The slowdown of receptor kinetics could further promote a reduction in frequency, given the increasing time required for receptor re-sensitization.

In conclusion, our study extends the reach of GABAergic dysfunction in the FXS mouse model, now to the mPFC—relevant to syndrome-related cognitive deficits. Prepubescent functional inhibition was stronger and inhibitory synaptic depression reduced, while in adolescence synaptic kinetics were prolonged, inhibitory frequency reduced, and receptor subunit expression deviated from control (Figure 8). These dynamic changes occurred during inhibitory and prefrontal circuit maturation, and can thus permanently alter downstream cognitive and behavioral circuits.

## Data Availability Statement

The datasets discussed in this article are available upon request.

## Ethics Statement

All procedures of animal handling and use for the experiments in this manuscript have been approved by the animal ethics committee of Vrije Universiteit Amsterdam.

## Author Contributions

All authors participated in the experimental design, analysis, and data interpretation aspects of the aforementioned research. Additionally, IK, RW, HL, and TH executed all electrophysiology experiments. Synaptosomal preparation and western blotting were executed by DR and SS. AL performed the Tsodyks-Markram Phenomenological Synaptic Transmission Model analysis. RM and HM provided resources, guidance, supervision, and support throughout this research. IK and RW wrote and revised the manuscript.

## Conflict of Interest

The authors declare that the research was conducted in the absence of any commercial or financial relationships that could be construed as a potential conflict of interest.
