# Supplementary figures and images for: Dysregulated Prefrontal Cortex Inhibition in Prepubescent and Adolescent Fragile X Mouse Model
Source: Front Mol Neurosci. 2020 May 26;13:88. doi: 10.3389/fnmol.2020.00088 (PMC7264168; doi:10.3389/fnmol.2020.00088)

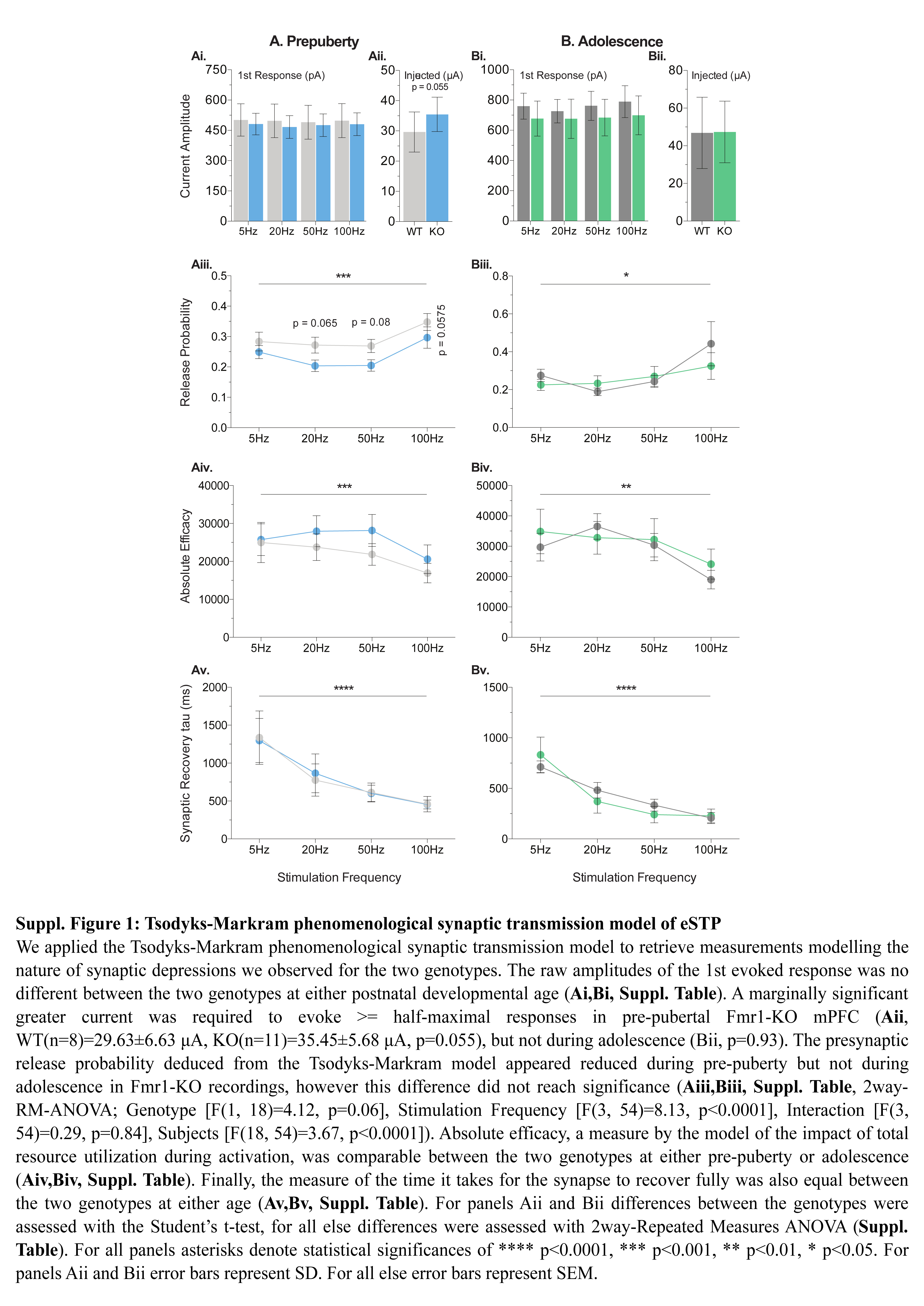

Supplement: Supplementary file 2 [file Image_1.jpg]

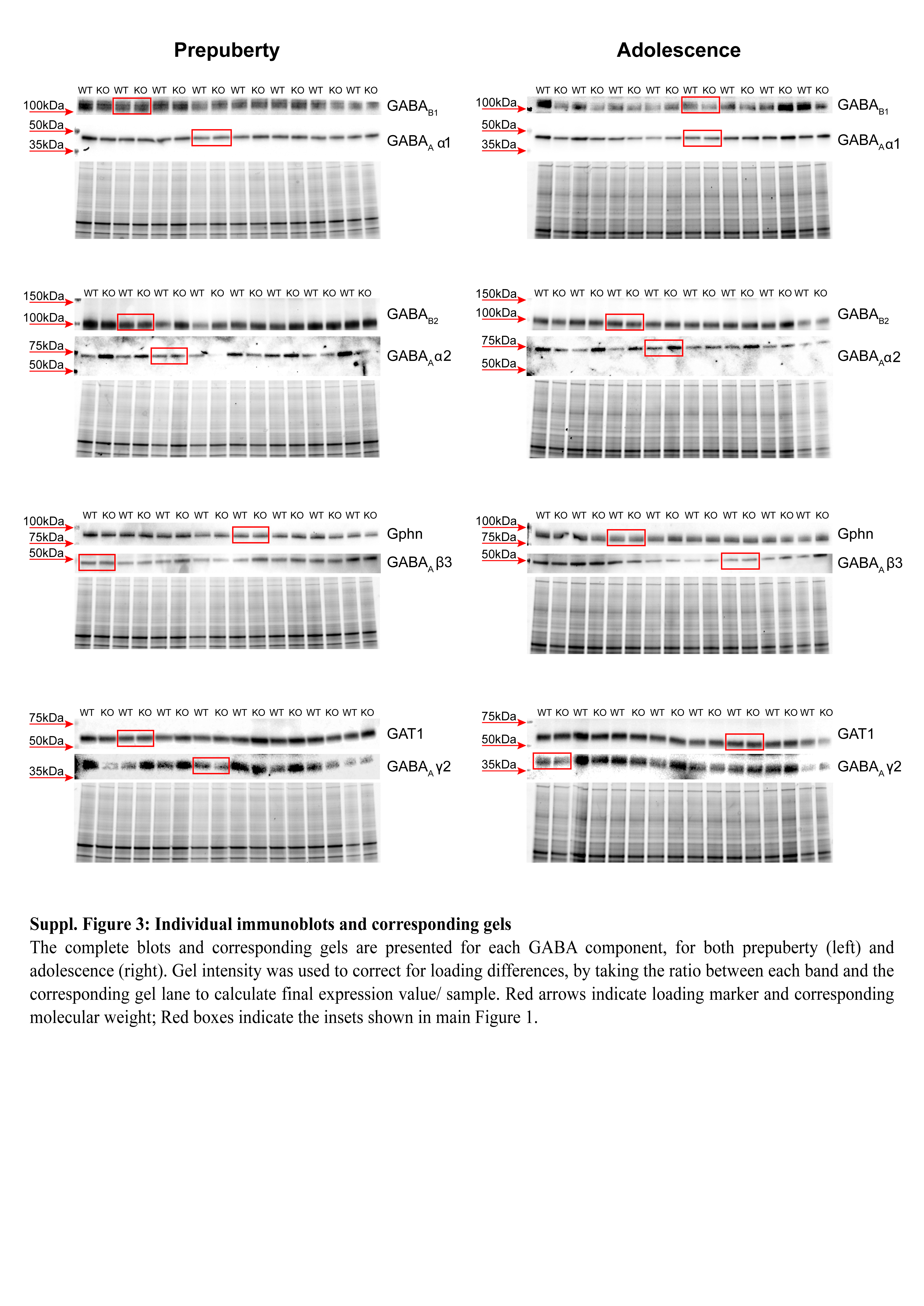

Supplement: Supplementary file 4 [file Image_3.jpg]

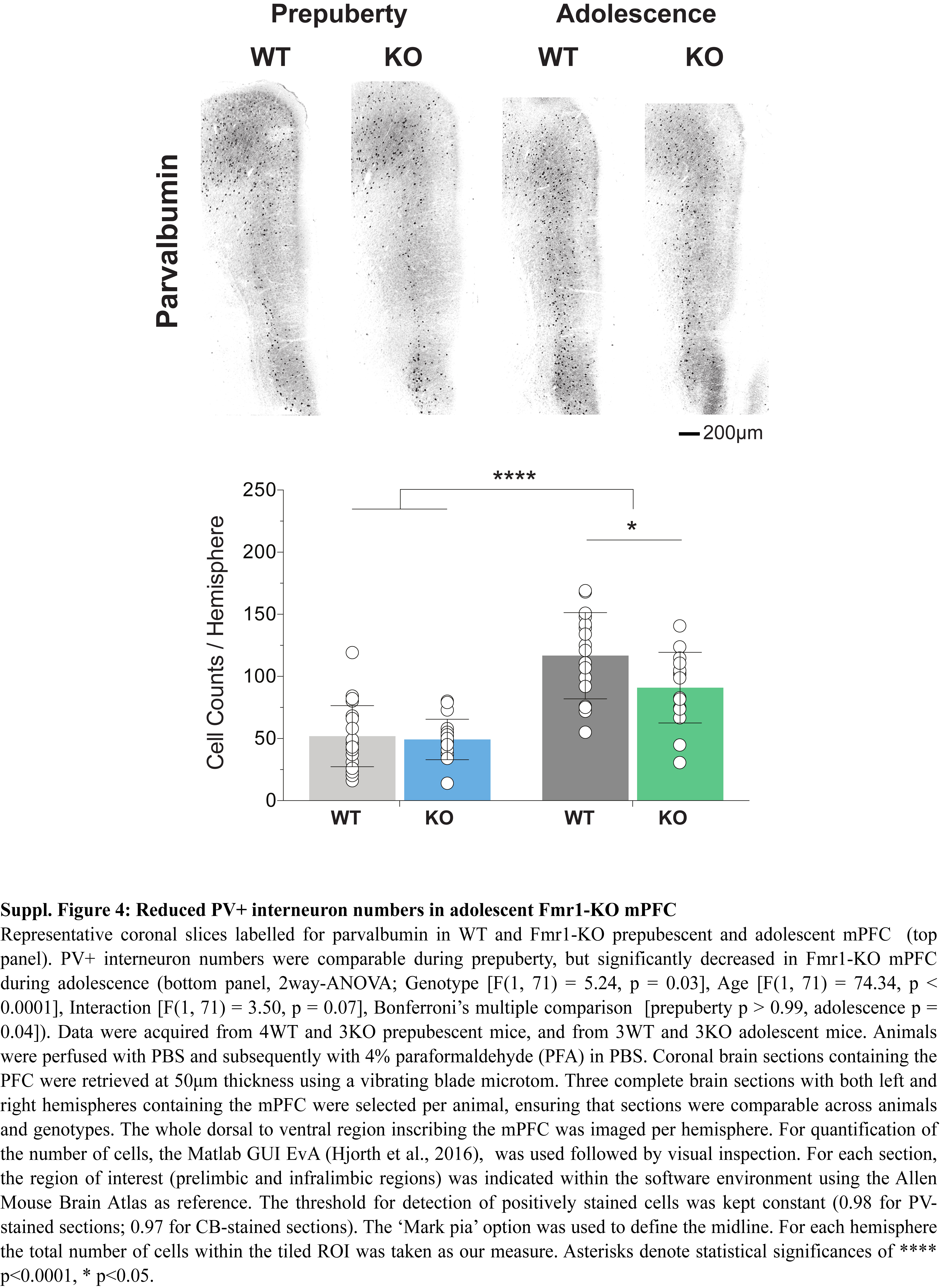

Supplement: Supplementary file 5 [file Image_4.jpg]
